# Host Response to *Coccidioides* Infection: Fungal Immunity

**DOI:** 10.3389/fcimb.2020.581101

**Published:** 2020-11-11

**Authors:** Anh L. Diep, Katrina K. Hoyer

**Affiliations:** ^1^Quantitative and Systems Biology, Graduate Program, University of California Merced, Merced, CA, United States; ^2^Department of Molecular and Cell Biology, School of Natural Sciences, University of California Merced, Merced, CA, United States; ^3^Health Sciences Research Institute, University of California Merced, Merced, CA, United States

**Keywords:** *Coccidioides immitis*, *Coccidioides posadasii*, Coccidioidomycosis, Valley fever, host pathogen interactions, fungal immunity

## Abstract

Coccidioidomycosis is a fungal, respiratory disease caused by *Coccidioides immitis* and *Coccidioides posadasii*. This emerging infectious disease ranges from asymptomatic to pulmonary disease and disseminated infection. Most infections are cleared with little to no medical intervention whereas chronic disease often requires life-long medication with severe impairment in quality of life. It is unclear what differentiates hosts immunity resulting in disease resolution versus chronic infection. Current understanding in mycology-immunology suggests that chronic infection could be due to maladaptive immune responses. Immunosuppressed patients develop more severe disease and mouse studies show adaptive Th1 and Th17 responses are required for clearance. This is supported by heightened immunosuppressive regulatory responses and lowered anti-fungal T helper responses in chronic *Coccidioides* patients. Diagnosis and prognosis is difficult as symptoms are broad and overlapping with community acquired pneumonia, often resulting in misdiagnosis and delayed treatment. Furthermore, we lack clear biomarkers of disease severity which could aid prognosis for more effective healthcare. As the endemic region grows and population increases in endemic areas, the need to understand *Coccidioides* infection is becoming urgent. There is a growing effort to identify fungal virulence factors and host immune components that influence fungal immunity and relate these to patient disease outcome and treatment. This review compiles the known immune responses to *Coccidioides* spp. infection and various related fungal pathogens to provide speculation on *Coccidioides* immunity.

## Introduction

Coccidioidomycosis is a fungal lung disease caused by inhalation of *Coccidioides immitis* and *Coccidioides posadasii*. It is a disease endemic to the Southwestern United States, Central America, and South America and is typically transmitted from the soil *via* wind (Johnson et al., 2014). In endemic regions of the American Southwest alone (California, Nevada, Utah, Arizona, and New Mexico) the estimated incidence is 122.7 cases per 100,000 (Benedict et al., 2019). Fungal arthroconidia enter the lungs and differentiate into a spherule state that replicates *via* endosporulation. Subsequent endospore rupture spreads the fungus, resulting in tissue damage and inflammation (Ternovoĭ and Golotina, 1977). Clinically, coccidioidomycosis is often misdiagnosed as pneumonia or lung cancer (Nguyen et al., 2013; Saenz-Ibarra et al., 2018). When the host immune system is unable to clear infection, it develops into a chronic state sometimes disseminating into other organs (Nguyen et al., 2013). In 60% of cases, infection remains asymptomatic or presents mild flu-like symptoms and is cleared by the host with little to no medical intervention (Saubolle et al., 2007). In 40% of cases, patients present moderate to severe flu-like symptoms that can become chronic. One percent of symptomatic cases develop severe disseminated infection (Saubolle et al., 2007).

In large part due to biosafety regulations, *Coccidioides* has been much less explored than other fungal pathogens. Though first reported in 1892, and with research dating back to the early 1900s, focus on the immune response against *Coccidioides* did not begin until the 1980s (Smith, 1940). A small but dedicated group of immunologists focus on fungal pathogens and host responses, but there is a critical need for further research into host responses to *Coccidioides*. There is little understanding of the immune events and players that contribute to disease resolution or chronic infection. Clinicians currently rely on symptom-based diagnosis, antibody-antigen assays, and imaging (chest x-rays and CT scans) to diagnose *Coccidioides*-infected patients, but these methods are limited in their ability to assess disease progression and host clearance capacity (Johnson et al., 2012; Wack et al., 2015). This review explores the initiation of innate immune responses and the development of adaptive immune responses to *Coccidioides*. Where gaps in *Coccidioides* knowledge exists, closely related fungal pathogens are used to extrapolate.

## Disease and Epidemiology

*Coccidioides* is endemic in regions with heavy intermittent rains along with the hot, arid summers (Johnson et al., 2014; Coopersmith et al., 2017; McCotter et al., 2019) It is found primarily in alkaline soils with high surface salinity (Elconin et al., 1964; Swatek, 1970; Lacy and Swatek, 1974). During wet, rainy months, filamentous threads composed of barrel-like subunits called arthroconidia expand within the soil. Environmental stresses, such as heat or digging, disrupts the soil and aerosolize the arthroconidia, making it airborne (McCotter et al., 2019). Infection occurs when arthroconidia are inhaled into the lungs and temperature and moisture differences trigger morphological change from arthroconidia to spherule to endospore (Johannesson et al., 2006). The fungal spherule develops into an endospore, capable of maturing and bursting to release more spores (Nguyen et al., 2013). As fungi develop, the host presents generic flu-like symptoms including headache, fever, body ache, coughing, and respiratory distress (Johnson et al., 2012; Wack et al., 2015).

*Coccidioides* infection also occurs in non-human animals, spanning across taxonomical classes from reptiles to mammals (Fisher et al., 2007; del Rocío Reyes-Montes et al., 2016). Animal carcasses are often found positive for *Coccidioides* while the surrounding soil environments test negative for the fungi (del Rocío Reyes-Montes et al., 2016). Originally, animals were believed to be accidental hosts, but genomic analysis indicates that *Coccidioides* code for several animal peptidases and lack enzymes associated with plant-decomposing fungi (Fisher et al., 2007; del Rocío Reyes-Montes et al., 2016; Taylor and Barker, 2019). This suggests that *Coccidioides* infected animals can act as fungal distributors, transporting the fungus from the initial infection site and upon animal death returning the fungus to new soil. Animal carcasses may also act as a protective, nutrient rich nursery for *Coccidioides* growth. Peptidase expression suggests that *Coccidioides* evolved methods to infect and thrive inside a protein rich environment, perhaps contributing to its success in surviving in harsh, alkaline environments. Wind and disturbance from other scavenging animals can then further disseminate the fungus driving human infection.

Agricultural, construction, and fieldwork in endemic regions are risk factors for fungal exposure. San Joaquin Valley (SJV) in California is an agricultural hub, supporting over 180,000 agricultural and 100,000 construction/labor jobs (Garcia and Young; Nicas, 2018). Solar energy field expansion puts solar-panel workers at risk for fungal exposure (Wilken et al., 2015; Laws et al., 2018). Legislative efforts in California have mandated *Coccidioides* risk education and safety protective equipment for at-risk workers in endemic areas (Salas, 2019). In Arizona, disease incidence increases with age, with those over the age of 70 experiencing the highest rate of disease (McCotter et al., 2019). Disease susceptibility for coccidiomycosis has been correlated with increasing age, with the elderly being much more susceptibility to infection and severe disease (Saubolle et al., 2007; Johnson et al., 2012; Nguyen et al., 2013; Wack et al., 2015). The high disease incidence in Arizona has been credited to the steady influx of new settlers over the last few decades and the increasing popularity of the state amongst retirees (McCotter et al., 2019).

Disease impact is further complicated by socioeconomic constraints. In California’s SJV, Hmong and Latino minorities make up a large percentage of field workers and soil-based laborers (Johnson et al., 2014). These populations tend to fall into the lowest wealth bracket with little to no access to healthcare, thus representing those with the least availability and opportunity to seek medical care, and the most exposed to *Coccidioides* (Mobed et al., 1992). Health care practioners working in the are often trained outside the endemic region and are initially unfamiliar with disease symptoms (Saenz-Ibarra et al., 2018). In 2007 in Arizona, only 50% of health care providers surveyed were confident in treating *Coccidioides* infection and only 21% correctly answered treatment questions (Chen et al., 2011). Since then, Arizona has implemented specialized coccidioidomycosis continuing medical education for in-state practioners, resulting in health care providers being twice as likely to answer treatment questions correctly compared to their untrained cohort. In California, outreach programs throughout endemic regions are providing disease awareness for physicians and patients, while legislative efforts aim to mandate coccidioidomycosis centric continuing medical education courses to provide specialized regional training (Salas, 2018).

## Innate Immunity

### Innate Detection and Immune Evasion

The lungs maintain many defense mechanisms to survey and eliminate airborne threats. Lung epithelial cells (LECs) secrete anti-microbial peptides, complement proteins, and defensins which enhance granulocyte activity and create a less hospitable environment for *Coccidioides* (Hernández-Santos et al., 2018). To survive, *Coccidioides* must successfully avoid detection from surveying and patrolling innate immune cells. Lung-resident macrophages, also known as alveolar macrophages, comprise up to 95% of pulmonary leukocytes and participate in early immune detection of pathogens and maintain the lung microenvironment (Wynn and Vannella, 2016). In *Aspergillus* infections, tissue-specific neutrophils are recruited by LECs and enter the lung early after infection due to β-glucan and chitin (Dubey et al., 2014). Innate leukocytes control early pathogen invasion *via* phagocytosis and production of reactive oxide and reactive nitrogen species (RNS) (Xu and Shinohara, 2017). β-glucan and chitin are conserved across many fungal species, including *Coccidioides*, so these molecules could interact with epithelial cells and aid in neutrophil recruitment. In cases where host immune responses cannot control infection, disease becomes chronic. Host responses sometimes control infections through granuloma formation in the lung as fungi is walled off instead of destroyed (Nguyen et al., 2013; Johnson et al., 2014; Wynn and Vannella, 2016).

**Figure 1:**
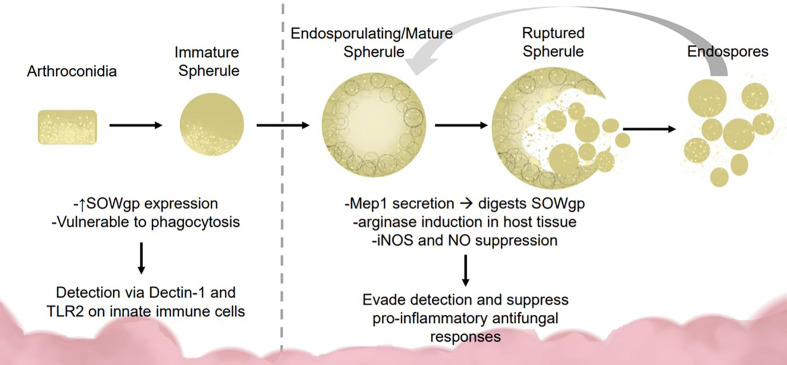
Fungal dimorphism presents challenges for immune detection and activation. Early infection: *Coccidioides* is vulnerable to immune detection during early infection due to the smaller size (2–5 μM) and SOWgp expression which is detected *via* Dectin-1 and TLR2 on innate immune cells. These interactions mediate clearance *via* phagocytosis and reactive oxide species production. Later infection: As *Coccidioides* sporulates, it secretes MEP1 which digests SOWgp from the fungal surface, hampering immune detection. Spherules induce arginase expression in host tissues, suppressing NOS/NO production *via* an unknown mechanism, contributing to immune suppression.

To survive lung defenses and evade innate immune responses, *Coccidioides* expresses virulence factors for immune evasion and survival. Inside the lung, arthroconidia express ornithine decarboxylase, an enzyme implicated during growth from arthroconidia to spherule state (Guevara-Olvera et al., 2000). During transition, the spherule internal cell wall segments bud off into endospores. Lifecycle transition allows vulnerable, easily phagocytosed, arthroconidia to develop into phagocytosis-resistant spherules (Hung et al., 2002; Gonzalez et al., 2011; Nguyen et al., 2013). Arthroconidia are vulnerable to RNS while mature spherules suppress nitric oxide species (NOS) and inducible NOS expression in macrophages (**Figure 1**) (Gonzalez et al., 2011). Mature spherules are too large for most host phagocytic activity, allowing *Coccidiodes* to evade early immune detection (Hung et al., 2002). *Coccidioides* induces host expression of arginase resulting in ornithine and urea production, important components for transition from arthroconidia to spherule (Hung et al., 2007).

In the spherule state, *Coccidioides* secretes metalloproteinase 1 (Mep1) which digests an immunodominant antigen spherical outer wall glycoprotein (SOWgp) on the fungal surface (**Figure 1**) (Hung et al., 2005). Phagocytotic granulocytes rely on pathogen associated molecular patterns such as SOWgp, thus Mep1 secretion prevents detection by innate immune cells (Hung et al., 2005). *Coccidioides* upregulates nitrate reductase during development, an enzyme that converts nitrate to nitrite, thereby enhancing *Coccidioides* survival in anoxic conditions, such as those found inside a granuloma (Johannesson et al., 2006). Early detection to inhaled fungus is critical for host response. Macrophages and neutrophils detect *Coccidioides* arthroconidia and immature spherules *via* receptors Dectin-1, Dectin-2, and Mincle interacting with SOWgp (Hung et al., 2002; Nguyen et al., 2013). Endothelial lung cells use these same receptors to regulate defensin secretion.

Toll-like receptors (TLRs) and c-type lectin receptors (CLRs) interact with major pathogen-associated molecular patterns to detect *Coccidioides* (Romani, 2004; Viriyakosol et al., 2008; Viriyakosol et al., 2013). Like most fungi, *Coccidioides* expresses β-glucans, chitins, and mannans in the outer cell wall (Nguyen et al., 2013). These cell components are recognized by a variety of TLRs and CLRs and elicit strong inflammatory responses from local immune cells. *Coccidioides* interactions with TLR2 and Dectin-1 on macrophages activate production of reactive oxide species (ROS) and inflammatory cytokines, such as interleukin-6 (IL-6) and tumor necrosis factor-alpha (TNFα) (Viriyakosol et al., 2008; Viriyakosol et al., 2013). There are no known nucleotide-binding oligomerization domain-like (NOD-like) receptors yet associated with *Coccidioides* detection.

In humans, polymorphisms in IFNγ/IL-12 signaling pathway result in a STAT1 gain of function mutations that associate with increased disease severity in *Coccidioides*, *Histoplasma*, and *Candida* infection (Sampaio et al., 2013). In disseminated *Coccidioides*, patients with severe disease were found to have a STAT3 mutation (Odio et al., 2015). STAT 3 mediates IL-23 signaling, critical for IFNγ, IL-12, and IL-17 production while STAT1 signaling induces Th1 cell differentiation in response to IL-12 to produce IFNγ; IFNγ, in turn, inhibits Th17 differentiation (Yeh et al., 2014). IL-12β1 receptor deficiency is associated with increased risk of disseminated coccidioidomycosis (Yeh et al., 2014). In chronic mucocutaneous candidiasis, gain of function mutations in STAT1 and STAT3 correlates to more severe disease and poor TH17 responses (Zheng et al., 2015). These observations suggest that STAT1 and STAT3 immune signaling is critical in host control of Th1/Th17 cytokine balance and is required for protection and *Coccidioides* fungal control.

In *Blastomyces dermatitidis* infection, LECs regulate collaborative killing between alveolar macrophages, dendritic cells (DC), and neutrophils (Hussell and Bell, 2014; Hernández-Santos et al., 2018). Upon LECs ablation, *B. dermatitidis* phagocytosis is reduced, and viable yeast numbers increase. Other data suggests that IL-1/IL-1R interactions regulate CCL20 expression in LECs. Chemokine CCL20 strongly recruits lymphocytes and weakly recruits neutrophils (Hernández-Santos et al., 2018). IL-1R-deficient mice express less CCL20 and lung Th17 cells are reduced, suggesting that IL-1/IL-1R signaling in LECs could regulate adaptive immune functions (Hernández-Santos et al., 2018). IL-1R is critical for vaccine induced resistance to *Coccidioides* infection *via* MyD88 induction of Th17 responses (Hung et al., 2014a; Hung et al., 2016a). Though it has not been explored, LECs could mediate early responses to *Coccidioides* through IL-1R, suggesting another innate immune cell role in anti-fungal responses within the lung tissues.

Alveoli structure likely helps shape local immune responses. Three dominant cell types exist within and around the alveoli structure: Type 1 and Type 2 pneumocytes (also known as alveolar epithelial cells, AECs), and tissue-resident alveolar macrophages (Guillot et al., 2013; Hussell and Bell, 2014). Type 1 pneumocytes (AECI) secrete IL-10 constitutively, which bind to IL-10R on alveolar macrophages to maintain an anti-inflammatory state. Type 2 pneumocytes (or AECII) express CD200 which interacts with CD200R on alveolar macrophage to inhibit pro-inflammatory phenotype (Guillot et al., 2013; Hernández-Santos et al., 2018). Alveolar macrophages express TGFβ-receptors that bind to pneumocyte-expressed αvβ6 integrin, tethering them in the alveolar airspace. In inflammatory conditions, AECIs upregulate TLRs and AECIIs increase SP-A and SP-D production (Guillot et al., 2013). These surfactant proteins are known to enhance pathogen opsonization and phagocytosis, and are capable of binding to *Coccidioides* antigen (Awasthi et al., 2004). *Coccidioides* infected mice expressed less SP-A and SP-D protein in their bronchial lavage fluid compared to uninfected and vaccinated controls, demonstrating the pathogen’s capability of altering the lung mucosa (Awasthi et al., 2004). AECII secreted production of surfactant proteins may be influenced by *Coccidioides* allowing fungal escape of phagocytosis and prolonged survival.

### Neutrophils

Neutrophils are the first responders and most abundant granulocytes in the immune system, making up 40%–70% of the total leukocyte population at homeostasis (Kolaczkowska and Kubes, 2013). Neutrophils destroy pathogens *via* phagocytosis, secretion of anti-microbial peptides, and extracellular traps, and provide signals for monocyte entry to sites of infection (Schaffner et al., 1986; Jain et al., 2016). In a 1:1 neutrophil to *Coccidioides* endospore interaction, human neutrophils readily phagocytose *Coccidioides* endospores and exhibit partial phagocytosis of larger spherules, coined “frustrated phagocytosis” (Lee et al., 2015). Neutrophils from healthy patients and chronic coccidioidomycosis patients exhibit similar neutrophil phagocytosis capabilities; however, high neutrophil presence is associated with chronic *Coccidioides* infection (Lee et al., 2015; Davini et al., 2018). This suggests that expanded neutrophil presence is detrimental for *Coccidioides* clearance perhaps due to their persistence into later stages of infection that may preclude other more effective responses (Davini et al., 2018). This in combination with neutrophil inability to fully phagocytose large endospores may make neutrophils ineffective, allowing prolonged fungal infection.

Neutrophil presence in tissue is typically associated with inflammatory tissue damage and pro-inflammatory cytokine expression such as IL-6 and IL-1β (Kolaczkowska and Kubes, 2013). Neutrophils follow C3a, C5a, IL-8, and IFNγ gradients toward sites of inflammation (Kolaczkowska and Kubes, 2013; Liu et al., 2017). However, chemoattractive molecules have limited stability and diffusion capabilities through tissues, suggestive that high neutrophil recruitment requires robust and/or steady expression of chemokines, which may also cause more inflammatory tissue damage. Until recently, it was believed that neutrophils migrate to infected tissue to mediate pathogen clearance and die within infected tissue after a few hours. Newer evidence suggests neutrophils re-enter circulation from the site of infection and may disseminate inflammation from the original recruitment site (Jain et al., 2016). For *Coccidioides* infection, this suggests a potential novel method for neutrophil dissemination of infection from the lungs where neutrophilia might promote disseminated disease. Depletion of neutrophils in *Paracoccidioides brasiliensis* infection results in decreased IFNγ and IL-17 with almost all infected mice succumbing to infection (Pino-Tamayo et al., 2016). This highlights the delicate balance of helpful versus harmful responses that pro-inflammatory innate immune cells play during disease clearance. In murine models of disseminated fungal infection with *Blastomyces*, *Aspergillus*, and *Candida*, neutrophils transdifferentiate into specialized hybrid neutrophil-DCs, and *in vitro*, neutrophil-DCs retain microbicidal, neutrophil-like function while also stimulating CD4+ T cell differentiation (Fites et al., 2018). This suggests a dual role for neutrophils in coccidioidomycosis, where too much neutrophil presence could contribute to disseminated disease and tissue damage, while some appropriate level response allows stimulation of adaptive immunity. *In vivo* examination and characterization of neutrophil-DC hybrids may provide a better understanding of innate immune cell influence on adaptive immune cell responses in *Coccidioides* infection. *In vivo* examination of neutrophil migration may unveil dissemination mechanisms, allowing for targeted therapeutics to prevent severe, disseminated coccidioidomycosis.

### Macrophages and Alveolar Macrophages

Macrophages engulf fungi and generate ROS and NOS that aid in degrading pathogens (Hussell and Bell, 2014). Fungicidal activity against *Coccidioides in vitro* by murine alveolar macrophages is enhanced in the presence of IFNγ (Beaman, 1987). IFNγ enhances and promotes phagocytosis, oxide species generation, pro-inflammatory cytokine production, and macrophage differentiation into the M1 phenotype for pathogen clearance and recruitment of other pro-inflammatory cells (Gentek et al., 2014). Pathogen recognition receptors on macrophages bind to targets for phagocytosis. Specifically, Dectin-1 and TLR2 interactions with *Coccidioides* facilitates clearance by macrophages by promoting oxide species and pro-inflammatory cytokine production (Viriyakosol et al., 2005; Tam et al., 2014).

Lung resident alveolar macrophages reside in air-exposed tissue compartments of the alveoli. Alveolar macrophages remove and clear particulates such as dust, pollutants, or airborne microorganisms in the respiratory mucosal surfaces (Lohmann-Matthes et al., 1994; Hussell and Bell, 2014). Alveolar macrophage-depleted mice challenged with *Aspergillus fumigatus* had higher fungal burden than wild-type mice (Gonzalez et al., 2011). When alveolar macrophages and DCs are ablated during *Aspergillus* infection, neutrophil infiltration increases (Lohmann-Matthes et al., 1994). This suggests alveolar macrophages and DCs may inhibit neutrophil recruitment during productive immunity to fungal lung infections.

**Figure 2:**
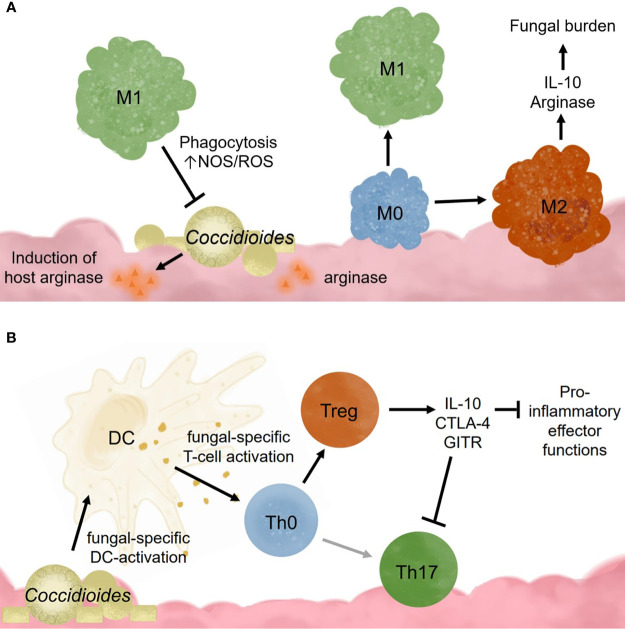
Innate immune cell responses to *Coccidioides* influence adaptive immune cell activation and effector functions. **(A)** *Coccidioides* may evade innate immune cell clearance and influence immune functions. **(B)** Dendritic cells are critical for activating adaptive responses and influencing adaptive immune cell population differentiation.

Alveolar macrophages also promote tolerance to commonly encountered lung antigens (Hussell and Bell, 2014). Resting alveolar macrophages closely resemble an M2 or alternatively activated macrophage (Hussell and Bell, 2014). It is theorized that these cells require cooperation between many receptors to override the basal anti-inflammatory, tolerogenic state found in the lungs (Wilken et al., 2015). Once activated, alveolar macrophages exhibit higher respiratory burst, phagocytotic capabilities, and inflammatory cytokine production (Lohmann-Matthes et al., 1994). These cells are regulated through interactions with IL-10R, CD200R, TGFβ-R, mannose receptors, and triggering receptors (TREM1 and TREM2), which all dampen proinflammatory signaling pathways (Hussell and Bell, 2014). Following inflammation caused by high viral infection, murine alveolar macrophages have decreased TLR2 responsiveness, low mannose receptor and high CD200R expression (Hussell and Bell, 2014). Acute inflammation seems to irreversibly change the overall alveolar macrophage responsiveness toward pathogen invaders. This has interesting implications for *Coccidioides* infection in patients with chronic inflammatory lung diseases such as asthma, chronic obstructive pulmonary disease (COPD), or high pollution exposure. In coccidioidomycosis, it is possible that M1 macrophages may be required for pathogen clearance, but instead become M2 due to signals from fungal factors (**Figure 2A**). Studies examining macrophages recruited following *Coccidioides* infection would help characterize the macrophage subtypes needed for fungal clearance. Such data may identify novel macrophage targets to treat *Coccidioides* infection by influencing macrophage differentiation.

### Eosinophils

Eosinophils are granulocytes associated with parasite infection, allergy, and asthma (Uhm et al., 2012). They make up 1%–3% of the leukocytes in the immune system and migrate to sites of inflammation *via* IL-5 chemotaxis (Yamaguchi et al., 1988; Uhm et al., 2012). Though not typically associated with an anti-fungal innate response, immunocompromised patients with allergic bronchopulmonary aspergillosis have high eosinophil lung infiltration during fungal infection, suggestive of maladaptive immunity (Chong et al., 2006). In chronic *Coccidioides*, infection correlates with peripheral blood eosinophilia (Harley and Blaser, 1994; Davini et al., 2018). Clinical observations from a *Coccidioides* case study highlight a correlation between asthma and poor fungal clearance, marked by high eosinophil lung infiltrate (Lombard et al., 1987). Increased IL-5 secretion in TNFα-deficient mice results in high eosinophil numbers and decreased IL-17A production, linking eosinophil changes to reduced adaptive responses (Fei et al., 2011). In acute *Paracoccidioides brasiliensis* infection, eosinophil recruitment is modest compared to uninfected mice but upon neutrophil depletion, eosinophil numbers increase significantly in the lungs (Pino-Tamayo et al., 2016). However, even with increased eosinophil presence, these mice still succumbed to infection with higher fungal burden. These data suggest that eosinophils could be recruited in the absence of neutrophils as a compensatory mechanism but unfortunately are not as protective as neutrophils.

Eosinophil presence in pulmonary coccidioidomycosis inversely correlates to neutrophil frequency (Lombard et al., 1987; Lee et al., 2015). In *Aspergillus*-allergy asthma murine models, lung DCs secrete TNFα which preferentially recruits neutrophils over eosinophils in the lung (Fei et al., 2011). This suggests local lung leukocytes influence immune cell recruitment and potentially control *Coccidioides* infection by establishing a different lung microenvironment (Fei et al., 2011). It is unknown whether asthma contributes to poor *Coccidioides* clearance, and asthma rates are high in current endemic regions. It could be that eosinophil and neutrophil recruitment varies between acute and chronic coccidiomycosis patients due to predisposed lung conditions such as allergies and asthma. Therefore, exploring pre-existing health conditions and disease progression may provide important clues to define effective clearance mechanisms.

### Dendritic Cells

DCs are professional antigen presenting cells, responsible for the initiation, regulation, and maintenance of adaptive immune responses (Desch et al., 2013). Lung-resident DCs must also maintain a balance between activation against invading pathogens and tolerance to continuous antigen exposure. DCs are adept at identifying fungal pathogens and promoting pathogen clearance, due to their powerful ability to activate naïve T lymphocytes (Romani et al., 2002). *Coccidioides* spherule recognition induces human DC maturation and activation resulting in elevated CD40 and CD80/CD86 (B7.1/B7.2) surface expression and heightened T lymphocyte stimulation (Dionne et al., 2006). DCs from healthy human patients, pulsed with *Coccidioides* spherule lysate, induce antigen-specific T cell activation (Richards et al., 2001).

There are many DC subsets with distinct functionality (Segura, 2016). Some promote inflammatory immune activation while others induce tolerance and tissue regeneration. Conventional DCs (cDC) versus monocyte-derived DCs (mDCs), exhibit opposing migration capacity to the lungs (Nakano et al., 2013). cDCs travel into the lung during homeostasis, whereas mDCs only migrate into lung tissue during active inflammation (Segura, 2016). Lung endothelial cells secrete IL-10, which maintains an anti-inflammatory, tolerogenic state and promotes anti-inflammatory DC function (Segura, 2016; Teitz-Tennenbaum et al., 2018). In IL-10 deficient mice infected with *Cryptococcus*, DCs upregulate inducible NOS expression and recruit neutrophils and M1 macrophages to the lungs during infection (Teitz-Tennenbaum et al., 2018). In contrast, IL-10-sufficient DCs express high arginase and CD206 (mannose receptor) which promotes tolerance within the lung by inducing IL-10 expression within endothelial tissues (Teitz-Tennenbaum et al., 2018). Maintaining an anti-inflammatory state within the lungs is critical for preventing unnecessary tissue damage to the delicate airspace architecture. Tolerance and anti-inflammatory signals are thus critical for ensuring that tissue-damaging inflammation does not occur unless pathogenic danger is imminent. Since DCs are responsible for controlling adaptive immune responses, understanding tissue-resident DCs and lung-microenvironmental influences is critical for understanding immune activation choices during pathogen detection and clearance.

DCs possess phagocytotic and pathogen clearance capabilities and act as professional antigen presenters to adaptive immune cells. Like macrophages that polarize into pro-inflammatory or anti-inflammatory subsets, DCs also exhibit polarization and DC polarization skews helper T cell differentiation toward specific subtypes (de Jong et al., 2005). *Cryptococcus neoformans* promotes an anti-inflammatory DC phenotype which suppresses inflammatory innate cells activation, allowing fungal persistence (Teitz-Tennenbaum et al., 2018). IL-10 blockade results in DCs with higher expression of costimulatory molecules and pro-inflammatory cytokines (Segura, 2016). In *Histoplasma capsulatum infection*, CD8α+ pro-inflammatory DCs were found to be critical for fungal clearance by inducing CD4+ T helper 1 (Th1) cells and CD8+ T cells (Lin et al., 2005).

DCs regulate T cell responses and immunological memory generation required for effective vaccines. Creating vaccines for dimorphic fungal pathogens is difficult as T lymphocytes recognize specific antigens and polymorphic fungal pathogens express different antigens throughout their life cycle. DCs have the unique challenge of presenting and mounting an effective immune response against *Coccidioides* regardless of morphological stage. Recent attempts at DC-based vaccinations show promising success in mouse models. Adoptive transfer of *Coccidioides*-antigen loaded DCs reduces murine fungal burden by live, virulent *Coccidioides* challenge (Awasthi, 2007). Disseminated coccidioidomycosis patient DCs loaded with T2K antigen overcame T cell anergy, driving T cell proliferation (Richards et al., 2002). This highlights a potential therapeutic where patients’ immune responses could be reactivated for fungal clearance. In murine vaccine studies, *Coccidioides* antigens delivered with glucan-chitin particles (GCP) effectively stimulate DC inflammatory responses. DCs loaded with GCP-antigen complex induce a mixed T helper 1 and T helper 17 response against *Coccidioides* infection, thought to be critical for effective fungal clearance (Hung et al., 2018a). These studies suggest that manipulating DC responses may be a viable route to creating vaccines that induce strong and specific immunity and may overcome pre-existing T cell anergy. The antigen subunit studies suggest effective DC activation requires multi-variant antigen interactions, and that multi-antigen vaccine therapies are promising strategies against *Coccidioides*.

## Introduction to Adaptive Immunity

Unlike innate immunity, adaptive immunity offers higher pathogen specificity, more powerful pathogen control mechanisms, and the ability to establish memory against future infections. Infection persistence implies a breakdown in immunity effectiveness or host feedback mechanisms protecting against damaging responses. To understand why chronic *Coccidioides* infections occur, we must first understand effective immunity to *Coccidioides*.

### B Cells

Protective immunity against *Coccidioides* is mediated predominantly by T cells (Hung et al., 2018a). *Coccidioides*-specific antibodies are observed in human and mouse studies, indicating that B cells also recognize and interact with *Coccidioides* antigen. IgG antibody is the prominent antibody isotype observed in humoral mediated responses to *Coccidioides* infection, indicative that class-switching occurs (Magee et al., 2005). Screening patients infected with *Coccidioides*, *Histoplasma*, and/or *Blastomycoses* with complement-fixing assays identified IgG1 as the predominant isotype generated.

Thirty days post-infection, mice have no neutralizing or complement-fixing antibodies in their sera against *Coccidioides* antigen and adoptive transfer of B cells from immunized mice into naïve mice does not confer protection (Beaman et al., 1979). In a contrasting study, T cell rich, B-cell deficient transfer conferred early protection, but the mice ultimately succumbed to disease 34 days post-infection (Magee et al., 2005). Mice that received whole splenocyte (mixed T cell, B cell, and other immune cell populations) transfers survived the longest. This could be due to the inclusion of B cells or other immune populations in the transfer. Vaccination with formalin fixed *Coccidioides* spherules and secondary intranasal challenge with live, virulent *Coccidioides* in BALB/c mice causes a marked increase in B cell-specific genes within whole lung tissue and generation of *Coccidioides*-specific serum antibodies (Magee et al., 2005). These studies suggest that B cells have a protective role in *Coccidioides* response and may even increase in frequency within the lung as suggested by bulk gene expression analysis data where B cell specific genes increased in expression in *Coccidioides* infected lung tissue compared to uninfected lung (Magee et al., 2005).

IgG generation requires CD4+ T cell-mediated class-switching and may explain the discrepancy between the B cell studies described above: T cells might provide initial protection, but without B cells there is no sustained antibody protection. Some *Coccidioides* antigens stimulate both T and B cell responses (Zhu et al., 1997). SOWgp is the best known immunostimulating *Coccidioides* antigen, eliciting a humoral response and innate immune cell activation (Hung et al., 2000). Disagreement around B cells in protective immunity against *Coccidioides* might be partially explained due to varied use of live-wildtype, live-attenuated, or formalin-fixed *Coccidioides* between studies, and the purity of cell populations utilized. *Coccidioides* is polymorphic and expresses multiple antigen types throughout its lifecycle, thus effective neutralizing antibodies and class-switching may be required at different stages of the immune response. High affinity antibody generation requires affinity maturation *via* somatic mutation, processes reliant on CD4+ T cell help. Considering all these data together, B cells likely contribute to adaptive and humoral immunity against *Coccidioides*, but further work is needed to definitively define the contribution to disease progression and control.

### T Cells and Effector Cytokines

Patients that recover from coccidioidomycosis with little to no medical intervention have polyfunctional T lymphocytes in circulation (Nesbit et al., 2010). Upon *Coccidioides* antigen stimulation, peripheral human CD4+ and CD8+ T lymphocytes secrete pro-inflammatory cytokines, such as IL-2, TNFα, and IFNγ (Nesbit et al., 2010). In humans, HIV and immunosuppression are risk factors for severe, disseminated infection and this risk is associated with decreased CD4+ T cell numbers (Saubolle et al., 2007; Johnson et al., 2012; Wack et al., 2015). In T cell-deficient mice, infection is severe, and effector T cell transfer protects mice against virulent infection (Fierer et al., 2006). CD4+ T cells from immunized C57BL/6 mice transferred into non-immunized CD40-deficient mice confer protection and prolong survival (Zhu et al., 1997). Vaccination with an attenuated *C. immitis* laboratory strain in CD4-deficient mice is protective, suggesting that CD8+ T cells can protect against *Coccidioides* (Fierer et al., 2006). No other studies show direct CD8+ T cells contribution to *Coccidioides* immunity. However, the CD8+ T cell study used an intraperitoneal infection, not intranasal delivery, so translation to pulmonary infection is unclear. In other fungal infections such as *Blastomyces* and *Histoplasma*, IL-17 producing CD8+ T cells provide protection and fungal clearance even in CD4-deficient mouse models (Nanjappa et al., 2012; Hung et al., 2016b). Overall, CD8α+ T cells may contribute to *Coccidioides* immunity, but further work is needed to characterize their anti-fungal mechanisms.

T cell differentiation into subsets allows targeted and tailored immune responses to different pathogen classes. For anti-fungal responses, T helper 1 (Th1) and T helper 17 (Th17) cells are especially critical in *Coccidioides* murine infections (Nanjappa et al., 2012). Loss of either of these T helper subtypes, or their associated cytokines, results in impaired immune responses and impaired fungal clearance. In human *Coccidioides* infection, Th17 cells are protective and acute coccidioidomycosis patients have high Th17 promoting serum cytokine levels (Davini et al., 2018). *In vitro Coccidioides* antigen stimulation of peripheral blood cells from acute disease patients yields robust IL-17 production (Hung et al., 2016b). *Coccidioides*-induced Th1 and Th17 cells secrete cytokines that mobilize innate immune cells to the site of infection, promote the activation and differentiation of immune cells, and induce anti-microbial peptides in endothelial cells (Hung et al., 2016b). This crossroad in innate immunity mediated by Th17 is observed in other fungal pathogens that infect mucosal tissues (Khader et al., 2009). Th1 cells make cytokines that enhance macrophage phagocytosis and ROS generation.

*Coccidioides*-resistant DBA/2 mice mount strong Th1 responses against *Coccidioides*, with increased serum IL-12 production (Magee and Cox, 1996). IL-12 administration to *Coccidioides*-susceptible BALB/c mice results in lowered fungal burden in lungs and spleen, whereas IL-12 blockage dramatically increases fungal burden across tissues, suggesting that IL-12 is protective against *Coccidioides* (Magee and Cox, 1996). DBA/2 mice express fully formed C-type lectin receptors, Dectin-1, unlike susceptible C56BL/6 mice with truncated Dectin-1. Dectin-1 is critical in fungal pathogen recognition and loss of dectin-1 correlates to increased fungal burden and decreased adaptive immune responses (Vautier et al., 2010) Dectin-1 interacts with *Coccidioides* β-1,3-glucans located in the outer cell wall and induces antibody class-switching and production, and CD8+ T cell activation (Viriyakosol et al., 2013). Dectin-1 binding to its ligand induces antigen presenting cell secretion of IL-1β, IL-23, IL-6, and TGFβ, cytokines necessary for Th17 cell differentiation. It is theorized that CARD9, an adaptor molecule downstream of Dectin-1 signaling, promotes intracellular signaling required for Th17 differentiation. CARD9-deficient mice are unable to clear pulmonary and subcutaneous infections with a highly virulent strain of *Coccidioides posadasii* (C735) (Hung et al., 2016a). These data emphasize the importance of fungal sugar pattern recognition receptor interactions for supporting Th1 and Th17 responses in adaptive immunity. In a multivalent vaccine study, CARD9 mediated Dectin-1 and Dectin-2 interactions were critical for establishing protection against *C. posadasii* infection (Campuzano et al., 2020). Mice not expressing CARD9, Dectin-1, or Dectin-2 all have significantly lower inflammatory cytokine responses and fail to mount Th17 responses within the lung. These data emphasize that though adaptive immune responses are critical, innate-associated receptors are required for establishing adaptive immunity, emphasizing that early innate interactions set the state for later adaptive responses.

C57BL/6 and BALB/c mice infected with an attenuated strain of *Coccidioides posadasii* (ΔT) have increased Th1 and Th17 frequencies and reduced fungal lung burden, further supporting the observation that these T helper responses are necessary for anti-fungal protection (Hung et al., 2016b). In other fungal pathogen studies, IL-17, IL-21, and IL-22 secretion by Th17 cells was vital for protection. IL-17 stimulates neutrophil and macrophage pro-inflammatory abilities and stimulates epithelial cells to secrete β-defensins (Khader et al., 2009). IL-21 acts as an autocrine regulator and promoter of Th17 proliferation, IL-22 induces host-secreted anti-microbial peptides, and TNFα promotes multiple proinflammatory pathways through NF-κβ and MAPK (Khader et al., 2009). These functions make Th17 cells and their effector cytokines very powerful against fungal pathogens. IL-1R deletion results in a significant decrease in Th17 numbers in *Coccidioides*-infected lungs, while Th1 numbers remain unchanged (Hung et al., 2014a). Lung Th17 numbers decrease in *Coccidioides*-infected IL-1R deficient mice relative to WT mice while Th1 numbers remain unchanged. In a human population study analyzing genetic susceptibility to *Blastomyces* infection, researchers found that IL-6 loss of function mutations increases susceptibility against *Blastomyces* infection (Merkhofer et al., 2019). IL-6 knock-out mice had lower Th17 responses and increased fungal burden within the lungs. These data emphasize the importance of Th17 responses against fungal infection while highlighting the complexity of cytokine networks needed to establish and regulate anti-fungal responses.

Th17 cells also participate in memory responses. In chronic pulmonary disease and fungal infection (*C. posadasii*, *H. capsulatum*, and *B. dermatitidis*), vaccine induced Th17 cells are sufficient for overcoming secondary challenge (Zelante et al., 2007). However, in *C. albican*s and *A. fumigatus* infection, Th17 cells are detrimental for fungal clearance, highlighting the variable role Th17 cells play in adaptive immunity. Th17 cells dampen neutrophil function and recruitment but also induce hyperinflammatory responses depending on the immune context (Zelante et al., 2007). There are two known Th17 subsets: pathogenic (GM-CSF producing) Th17 and non-pathogenic (IL-10 producing) Th17 (El-Behi et al., 2011; Bystrom et al., 2019). While advances have been made in defining effector T cell functions during chronic fungal infection, much more work is needed to understand how chronic inflammation alters function. This may explain why Th17 cells are critical for memory responses, host survival and fungal clearance in some fungal infections but damaging and ineffective in others.

In a *Coccidioides* vaccine study, loss of Th17 immunity increased infection susceptibility while loss of Th1 and Th2 immunity did not, implying that Th1 and Th2 cells are not critical for protection (Hung et al., 2011). The underlying mechanisms for this protection are less clear. For example, Th2 cells can promote alternatively activated macrophages which secrete collagen and assist in tissue repair. Alternatively activated macrophages are often recruited to sites of infection where their collagen production assists in establishing granulomatous structures. Virulent and avirulent *Coccidioides* strains form granulomas *in vivo*, and while morphologies have been characterized. we do not know what cells are recruited to the granuloma, what signals form and maintain the granuloma structure, nor details on the immune microenvironment within the granuloma interior (Narra et al., 2016). Exploring granuloma immunity is imperative for understanding infection chronicity as *Coccidioides* infection often presents with granuloma formation. Such knowledge could inform diagnosis and provide markers that distinguish fungal granulomas from cancer nodules and bacterial granulomas.

CD4+ T cell subset frequency is also correlated with infection outcome in human patients. In a pediatric coccidioidomycosis study, high regulatory T cell (Treg) frequency correlated with chronic disease (Davini et al., 2018). Patients with a similar fungal infection, *Paracocidioides brasiliensis*, have a higher %Tregs than healthy controls, and the Tregs are more suppressive (Odio et al., 2015). In mouse models, Treg depletion after infection with *Paracoccidioides brasiliensis* resulted in decreased fungal burden and enhanced survival (Galdino et al., 2018). Chronic *Coccidioides* patients also express heightened serum IL-10 cytokine levels. IL-10 is an effector cytokine used by Tregs to suppress immune activation and reduce inflammation (Cavassani et al., 2006). In the absence of IL-10, susceptible mice develop a protective immune response and lasting immune memory against virulent *Coccidioides posadasii* (Hung et al., 2014b). Tregs regulate immune responses by controlling immune cell activation to prevent hyperinflammatory, damaging responses (Montagnoli et al., 2002; Wing et al., 2008). The mechanisms underlying Treg association with chronic *Coccidioides* disease outcome are unknown. Elevated Treg frequency may block effector responses by overwhelming effector cells, Tregs may be more suppressive, or Tregs may develop at the expense of Th17 responses (Davini et al., 2018). Treg and Th17 differentiation are inversely regulated; the signals required for Treg development block Th17 differentiation (Khader et al., 2009; Yeh et al., 2014). Under inflammatory conditions Tregs can lose regulatory, and gain effector, functions promoting chronic inflammation (Wing et al., 2008). *Coccidioides*-resistant DBA/2 mice have lower lung Treg frequency that produce less IL-10 upon stimulation *ex vivo* compared to susceptible mice (**Table 1**) (Fierer et al., 1998; Viriyakosol et al., 2008; Hung et al., 2014b). These studies suggest a detrimental role for Tregs in *Coccidioides* clearance and emphasize a need to explore their function and plasticity during *Coccidioides* infection. It is unlikely that gain of effector functionality is occurring during chronic *Coccidioides* infection as T helper cells enhance fungal control. Further work must be done to determine whether Treg presence and functionality direct adaptive immune responses and reduce *Coccidioides* control.

**Table 1:** Resistance to *Coccidioides* infection in specific strains could be due to immune cellularity differences.

Mouse Background	Susceptibility to *Coccidioides* Infection	Alveolar Macrophage Freq.	Lung-resident DC Freq.	Respiratory Leukocyte TNFα production	Respiratory Leukocyte IL-10 production	Lung CD4+CD25+FOXP3+ (Treg) Freq.	Reference Numbers
**C57BL/6**	Susceptible	Base	Base	Base	Base	Base	(Fierer et al., 1998; Viriyakosol et al., 2008; Viriyakosol et al., 2013; Hung et al., 2014b)
**BALB/C**	Susceptible	No Δ	No Δ	↑↑	↑↑	↑↑	(Fierer et al., 1998; Viriyakosol et al., 2008; Hung et al., 2014b)
**DBA/2**	Resistant	No Δ	No Δ	No Δ	↓	↓	(Fierer et al., 1998; Viriyakosol et al., 2008; Viriyakosol et al., 2013; Hung et al., 2014b)

The base frequency of cells or cytokine production is in reference to C57BL/6 mice; denoted changes are relative to this strain. Cytokine production was measured post stimulation ex vivo. No Δ, no change from base level; ↑↑, higher than base; ↓, less than base.

**Figure 3:**
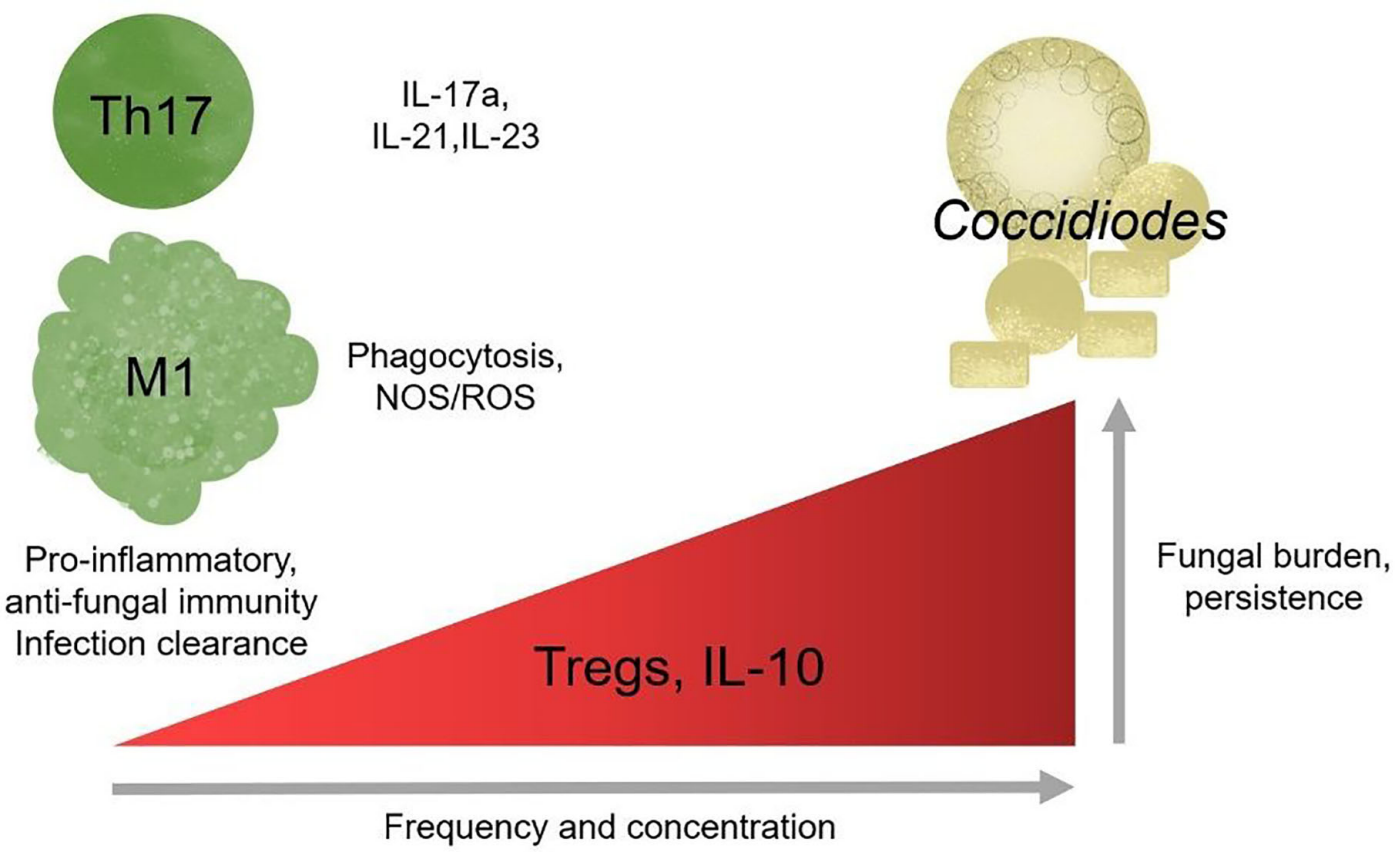
Regulatory responses correlate with chronic disease outcome. Th17 cells and pro-inflammatory responses are protective against *Coccidioides* and loss of responses results in increase susceptibility in mice. Modulating effector and regulatory responses might be a potential therapy for chronic infection.

In better studied models of chronic inflammation, such as chronic viral infections and cancer, T cells upregulate inhibitory receptors, reducing effector function, in a process termed exhaustion (Wherry and Kurachi, 2015). Chronic antigen exposure is sufficient to drive T cells toward exhaustion, often marked by elevated PD-1 and PD-L1 surface expression (Wherry and Kurachi, 2015). There is very little work examining T cell exhaustion in the context of chronic fungal infections, but existing works that look at chronic *Candida* sepsis patients supports the general observation that exhaustion is detrimental to host health and fungal clearance (Lázár-Molnár et al., 2008; Spec et al., 2016). In *Histoplasma* infections, mice lacking PD-1/PD-L1 survive severe infection while wild-type do not (Lázár-Molnár et al., 2008). *Candida* sepsis patients have elevated circulating PD-1/PD-L1 high T cell frequency (Spec et al., 2016). In paracoccidiomycosis, T cells overexpress PD-1 suggestive of early stage exhaustion during chronic infection (Cacere et al., 2008). It is unknown whether chronic coccidioidomycosis may occur due to T cell exhaustion and maintenance by inappropriately mounted regulatory responses; if so, immune checkpoint blockade could benefit patients with fungal induced T cell exhaustion and promote fungal clearance (**Figure 3**).

## Vaccinations and Immune Memory

There is currently no fungal vaccine clinically available for humans. Fungal pathogens are eukaryotic, sharing many conserved molecules with humans, making drug and vaccine targeting highly difficult without targeting human cells (Johannesson et al., 2006; Nguyen et al., 2013). *Coccidioides* is dimorphic; the soil and host forms express different surface molecules (Johannesson et al., 2006; Saubolle et al., 2007; Nguyen et al., 2013; Johnson et al., 2014). Thus, effective vaccine strategies must include components that would elicit both a strong and effective immune response without selecting temporal life cycle antigen targets. A *C. albicans* vaccine, NDV-3A, in stage 1b/2a clinical trial that shows promise in protecting patients from recurring vulvovaginal candidiasis (Alqarihi et al., 2019). NDV-3A success demonstrates the major strides made in fungal vaccine research and highlight how much further we have to go. The field has made remarkable progress in identifying adjuvants, antigens, and target cells for vaccine therapies, but further work must be done to demonstrate reliable efficacy between animal models and human use, especially in less studied fungal pathogens such as *Coccidioides*.

Several labs have generated live, attenuated strains of *Coccidioides* that successfully confer protection against secondary challenges with virulent, wild-type *Coccidioides* in susceptible mouse strains (Xue et al., 2009; Hung et al., 2011). The attenuations hinder fungal replication within the host, pausing *Coccidioides* growth at the spherule state. Mice vaccinated with attenuated *Coccidioides* strain ΔT mount Th1 and Th2 effector responses and survive longer than non-vaccinated littermates during post-secondary challenge (Xue et al., 2009). Protein component vaccination with antigen2/PRA (a deglycosylated, proline-rich antigen expressed in *Coccidioides* spherules) is protective when administered subcutaneously or intranasally in susceptible mice (Shubitz et al., 2002). However, this protection decreases at higher fungal doses and only intranasal administration of antigen2/PRA confers protection to both C57BL/6 and BALB/C mice. This component vaccine prolongs survival, but immune cell response to antigen2/PRA has yet to be characterized (Shubitz et al., 2002). Following up on antigen2/PRA as a promising vaccine strategy, mouse bone marrow derived DCs presenting the antigen2/PRA epitope, were intranasally transferred into mice and their immune response analyzed (Awasthi et al., 2019). Lymphocytes and leukocytes from immunized mice express more IFNγ, IL-4, and IL-17 compared to mice that receive control DCs. While this study did not challenge mice with *Coccidioides* post-immunization, it demonstrates the possibility of immune-cell transfer as a vaccination strategy. ΔT vaccination studies show a mixed Th1, Th2, and Th17 response in immunized mice and conferred protection in susceptible C57BL/6 mice (El-Behi et al., 2011). This vaccine’s success emphasizes adaptive immunity’s importance in protection. Maize provides more efficient generation of antigen2, and a maize-produced subunit vaccine along with the previously discussed glucan-chitin particle delivery method protected mice against *Coccidioides* and yielded lower fungal burden (Hayden et al., 2019).

The most effective vaccines include adjuvants and additives that enhance immunity and memory. Most notably, adding an agonist of human fragment C5a enhances vaccine immunity against *Coccidioides* infection in mice (Hung et al., 2012). Human C5a added during ΔT vaccination in BALB/c mice, causes heightened Th1 and Th17 responses and circulating effector cytokines; furthermore, humanC5a/ΔT vaccinated mice express higher titers of IgG1 and IgG2 specific to *Coccidioides* and survive longer compared to BALB/c mice vaccinated with ΔT alone (Hung et al., 2012). Ablating IL-10 with ΔT vaccination increases survival and protection against virulent *Coccidioides* infection and increases recall protection post infection (Hung et al., 2014b). These studies further reinforce Th1/Th17 cells in immunity against *Coccidioides* while IL-10 plays a negative role in clearance. This suggests *Coccidioides* immunity requires robust pro-inflammatory responses, while immunosuppression leads to infection persistence.

To complement vaccine research, we need to understand the memory responses vital for sustained immunity. While memory responses have been observed in *Coccidioides* vaccine experiments, tissue-resident memory, effector memory, and central memory subtypes have not been characterized in *Coccidioides* infection. Memory research has made great strides in characterizing cytokine recall responses, but further characterization is required to identify the source of the memory cells. In *Candida* infection, skin-resident memory T cells remain after infection and reactivate upon reinfection (Iannitti et al., 2012). Since many fungal infections occur within mucosal tissues and effective clearance requires tissue specific responses, fungal vaccine development could benefit from exploring tissue-specific memory (Iannitti et al., 2012). In tuberculosis, a bacterial infection associated with granuloma formation, intranasal vaccination with attenuated mycobacterium induces protective CD4+ and CD8+ T cells and mucosa associated lymphoid responses (Walrath et al., 2005). Further analysis revealed tissue resident memory responses from lung parenchyma are critical for protection against virulent mycobacterium (Walrath et al., 2005; Sakai et al., 2014). This validates intranasal delivery as an effective method of vaccination for stimulating tissue resident memory development. While tuberculosis is a bacterial infection, similarities between tuberculosis granulomas and coccidioidomycosis granulomas highlight tissue-resident memory responses as a key for long-lasting immunity in chronic lung infections. Coccidiomycosis starts as a localized respiratory infection, so tissue-resident memory could be critical for providing long lasting protection and warrants further study.

Current work suggests fungal sugar receptors are highly important for the development of anti-fungal recall responses (Shubitz et al., 2002; Cavassani et al., 2006; Hung et al., 2018b). However, much of what we know about memory immunity to *Coccidioides* has used live-attenuated laboratory strains, fungal-derived antigens, and fungal sugar adjuvants to achieve protective immune responses. Characterizing the memory response to live, whole, virulent *Coccidioides* might help to define how memory is generated, or perhaps blocked from generation, in natural infections. Such immunological questions would aid vaccine development, providing a broader context of *Coccidioides*-specific challenges for effective memory responses where our more defined, specific studies are lacking Together, these studies would allow for an elevated understanding of *Coccidioides* immunity as a whole, generating the specific antigen knowledge for vaccine development and broad characterization for better patient treatment.

## Conclusion: Clinical Significance and Urgency

Valley fever research is at a critical point. California’s highest endemic region for Valley fever, SJV, has one of the lowest physicians to citizen ratios, adding a barrier to health care access (Petterson et al., 2012). Drugs used to treat chronic Valley fever cause debilitating side-effects in patients such as, but not limited to, headaches, lethargy, seizures, severe hair lost, extreme exhaustion, and nerve pain (Saubolle et al., 2007; Saenz-Ibarra et al., 2018). Due to its generic flu-like symptoms, it is often misdiagnosed as other respiratory infections, leading to late stage diagnosis when more severe symptoms of chronic infection manifest. Since disease clearance or progression determinants are unknown, early diagnosis is invaluable for planning patient treatment. Susceptibility marker identification would help determine those vulnerable to chronic disease. Understanding how the innate and adaptive immune system responds to Valley fever is necessary for optimal diagnoses, treatments, disease progression predictions, and vaccine development. Such studies will inform accurate diagnoses and perhaps provide novel drug targets for therapy. It is unclear whether *Coccidioides* infection becomes chronic because i) the host has high regulatory responses and thus suppresses pro-inflammatory responses, ii) *Coccidioides* is manipulating and influencing host innate immunity to block inflammatory responses, which, in turn, promotes ineffective helper responses, or iii) a combination of host and pathogen influences (**Figures 2A, B**). These observations emphasize the importance of a pro-inflammatory response and suggest that inhibition of pro-inflammatory players promote infection persistence. Tregs seem especially pertinent given supportive pediatric patient and depletion data in *Paracoccidioides* (Felonato et al., 2012). In some settings, Tregs retain their suppressive function yet cannot control T effector responses. This is because T effector cells become refractory to Treg suppression. While this has not been assessed, it is possible that T effector cells could become more sensitive to suppression by Tregs, reducing their effector functionality. Characterizing innate immune cells activation and recruitment of adaptive responses may define how *Coccidioides* eludes clearance and shed light on the role of pro-inflammatory and regulatory responses in disease progression (**Figure 3**).

Until the last 30 years, fungal pathogens have not been as well studied as their viral or bacterial counterparts. The field is at a critical and exciting time as we work together to close gaps in fungal pathogen host immune knowledge. Climate change models predict *Coccidioides’* endemic regions will spread to the American Midwest by 2050. Thus *Coccidioides* is anticipated to spread significantly beyond the current regions of endemicity (Coopersmith et al., 2017; Gorris et al., 2019). Though severe disease is rare, it is unknown what factors indicate infection susceptibility and disease progression. As antifungal resistance, fungal disease frequency, and regions of endemicity increase, the urgency and need for effective vaccines and better therapeutics also rise. Due to current lack of effective treatment options for chronic disease, the inability to determine likelihood of disease progression toward chronicity at time of diagnosis, and the growing spread of the endemic region, there is a dire need to fully understand host immune response for improved diagnoses and treatment.

## Author Contributions

AD: conceptualization, literature evaluation, original draft writing, and generated and visualized figures. KH: conceptualization, writing and review, visualization, funding acquisition, and supervision. All authors contributed to the article and approved the submitted version.

## Funding

This work was supported by the University of California (UC) Office of the President grant VFR-19-633952 and UC Multicampus Research Programs and Initiatives grant 17-454959, and a private donation from Robert Hayden and Betty Dawson.

## Conflict of Interest

The authors declare that the research was conducted in the absence of any commercial or financial relationships that could be construed as a potential conflict of interest.
